# Genome analysis of a novel Group I alphabaculovirus obtained from *Oxyplax ochracea*

**DOI:** 10.1371/journal.pone.0192279

**Published:** 2018-02-01

**Authors:** Jun Wang, Dianhai Hou, Qianran Wang, Wenhua Kuang, Lei Zhang, Jiang Li, Shu Shen, Fei Deng, Hualin Wang, Zhihong Hu, Manli Wang

**Affiliations:** 1 State Key Laboratory of Virology, Wuhan Institute of Virology, Chinese Academy of Sciences, Wuhan, China; 2 School of Bioscience and Technology, Weifang Medical University, Weifang, China; Swedish University of Agricultural Sciences, SWEDEN

## Abstract

*Oxyplax ochracea* (Moore) is a pest that causes severe damage to a wide range of crops, forests and fruit trees. The complete genome sequence of Oxyplax ochracea nucleopolyhedrovirus (OxocNPV) was determined using a Roche 454 pyrosequencing system. OxocNPV has a double-stranded DNA (dsDNA) genome of 113,971 bp with a G+C content of 31.1%. One hundred and twenty-four putative open reading frames (ORFs) encoding proteins of >50 amino acids in length and with minimal overlapping were predicted, which covered 92% of the whole genome. Six baculoviral typical homologous regions (*hr*s) were identified. Phylogenetic analysis and gene parity plot analysis showed that OxocNPV belongs to clade “a” of Group I alphabaculoviruses, and it seems to be close to the most recent common ancestor of Group I alphabaculoviruses. Three unique ORFs (with no homologs in the National Center for Biotechnology Information database) were identified. Interestingly, OxocNPV lacks three auxiliary genes (*lef7*, *ie-2* and *pcna*) related to viral DNA replication and RNA transcription. In addition, OxocNPV has significantly different sequences for several genes (including *ie1* and *odv-e66*) in comparison with those of other baculoviruses. However, three dimensional structure prediction showed that OxocNPV ODV-E66 contain the conserved catalytic residues, implying that it might possess polysaccharide lyase activity as AcMNPV ODV-E66. All these unique features suggest that OxocNPV represents a novel species of the Group I alphabaculovirus lineage.

## Introduction

Baculoviruses are known to infect a wide variety of insect hosts and play important roles in regulating many insect populations in nature. They have been widely used as environmentally safe agents for pest control. In addition, baculoviruses provide efficient expression systems for the production of recombinant proteins in insect cells, as well as promising vectors for gene therapy [1, 2]. These applications greatly facilitate fundamental studies of baculoviruses.

To date, more than 600 baculoviruses have been reported to be isolated from different insect species, and 87 of them have had their whole genomes sequenced [3]. Baculovirus genomes consist of circular, double-stranded DNA ranging from 80 to 180 kb [4, 5]. Based on phylogenetic analysis, the family *Baculoviridae* is classified into four genera: *Alphabaculovirus* (lepidopteran-specific nucleopolyhedroviruses [NPVs]), *Betabaculovirus* (lepidopteran-specific granuloviruses [GVs]), *Gammabaculovirus* (hymenopteran-specific NPVs) and *Deltabaculovirus* (dipteran-specific NPVs) [6, 7]. The *Alphabaculovirus* genus can be divided into two groups, Groups I and II [8–10]. Group I alphabaculoviruses are characterized by their use of GP64 as their envelope fusion protein (EFP), while Group II alphabaculoviruses, most betabaculoviruses (except a newly discovered Diatraea saccharalis granulovirus, which contains both *gp64* and *f*) and deltabaculoviruses exploit F protein as their EFP [11, 12]. Group I alphabaculoviruses are further divided into two clades based on phylogeny, “a” and “b” [13]. Despite the diversity in gene content and organization of baculovirus genomes, a set of 38 core genes are conserved across their genomes, which play important roles in the viral life cycle [14, 15]. In addition, 22 genes are conserved in all sequenced lepidopteran baculoviruses (alpha- and betabaculoviruses) and 11 genes (including *gp64*) are only present in Group I alphabaculoviruses [16].

*Oxyplax ochracea* (Moore) (Lepidoptera: Limacodidae) causes economic losses related to orange, tea, and tea-oil trees. It is widely distributed in East, South and Central China, as well as in Indonesia, India, Thailand and Sri Lanka [17]. In China, the insect has two generations each year [18]. Oxyplax ochracea nucleopolyhedrovirus (OxocNPV) is a natural pathogen of *O*. *ochracea*. It was first isolated in 1989 from *O*. *ochracea* larvae that exhibited the typical symptoms of baculovirus infection in an orange field in Zhuxi, Hubei Province [18]. Electron microscopy showed that OxocNPV is a single-nucleocapsid nucleopolyhedrovirus (SNPV), with occlusion bodies (OBs) containing singly packaged virions [18]. In this study, the complete genome sequence of OxocNPV was determined and analyzed. Phylogenetic analysis suggested that this virus is a novel Group I alphabaculovirus and seems to be closely linked to the most recent common ancestor of these viruses.

## Results and discussion

### Sequencing and characterization of OxocNPV genome

**Fig 1:**
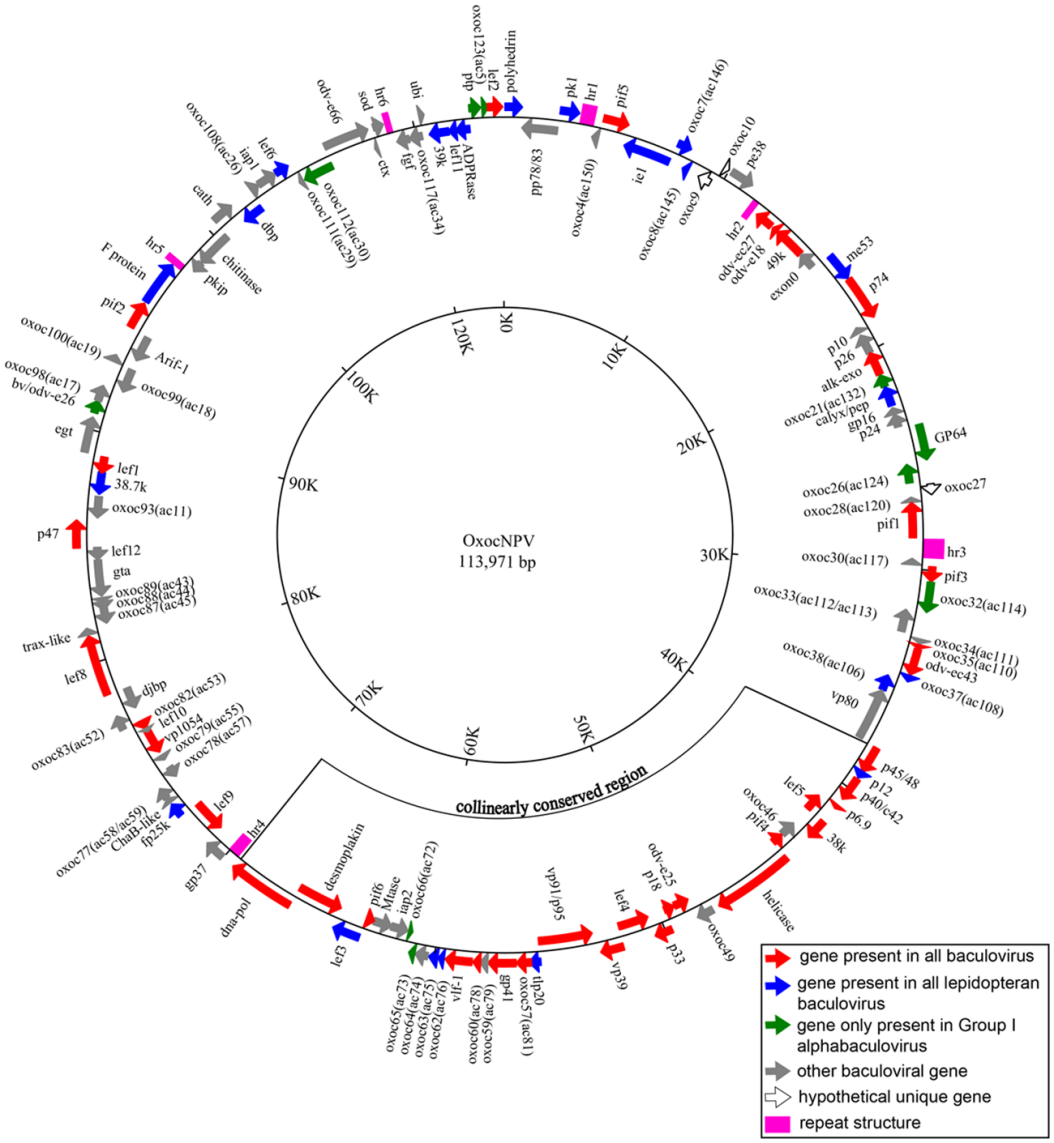
Circular map of OxocNPV genome. ORFs are indicated by arrows. The direction of the arrows indicates the orientation of gene transcription. The colors represent gene types: red for core genes, blue for lepidopteran baculovirus conserved genes, green for Group I unique genes, and gray for other baculoviral genes. Open arrows represent hypothetical unique genes of OxocNPV. *Hr*s are represented by pink square boxes. The collinear region conserved in lepidopteran baculoviruses is also indicated.

The genome of OxocNPV was assembled from 71,240 high-quality Roche 454 sequencing reads with an average coverage of 249X. Uncertain regions were confirmed by PCR amplification and Sanger sequencing. The complete genome sequence and annotation information were submitted to GenBank (accession number: MF143631). In summary, the complete circular OxocNPV genome is 113,971 bp in length, with a G+C content of 31.1%. In total, 124 putative open reading frames (ORFs) that potentially encode proteins of >50 amino acids (aa) in length were predicted, covering 92% of the whole genome. Among them, 61 ORFs were in the forward orientation and 63 were in the reverse orientation. The *polyhedrin* gene was assigned as the first ORF according to tradition. The 38 baculovirus core genes (Fig 1, red), 22 lepidopteran baculovirus conserved genes (Fig 1, blue), 10 Group I alphabaculovirus unique genes (Fig 1, green) and 51 baculovirus common genes (Fig 1, gray) were annotated using Basic Local Alignment Search Tool (BLAST) comparisons. In addition, three genes were classified as OxocNPV unique genes as no homologs were found in the National Center for Biotechnology Information (NCBI) database (Fig 1, open arrows).

### Phylogenetic analysis of OxocNPV

**Fig 2:**
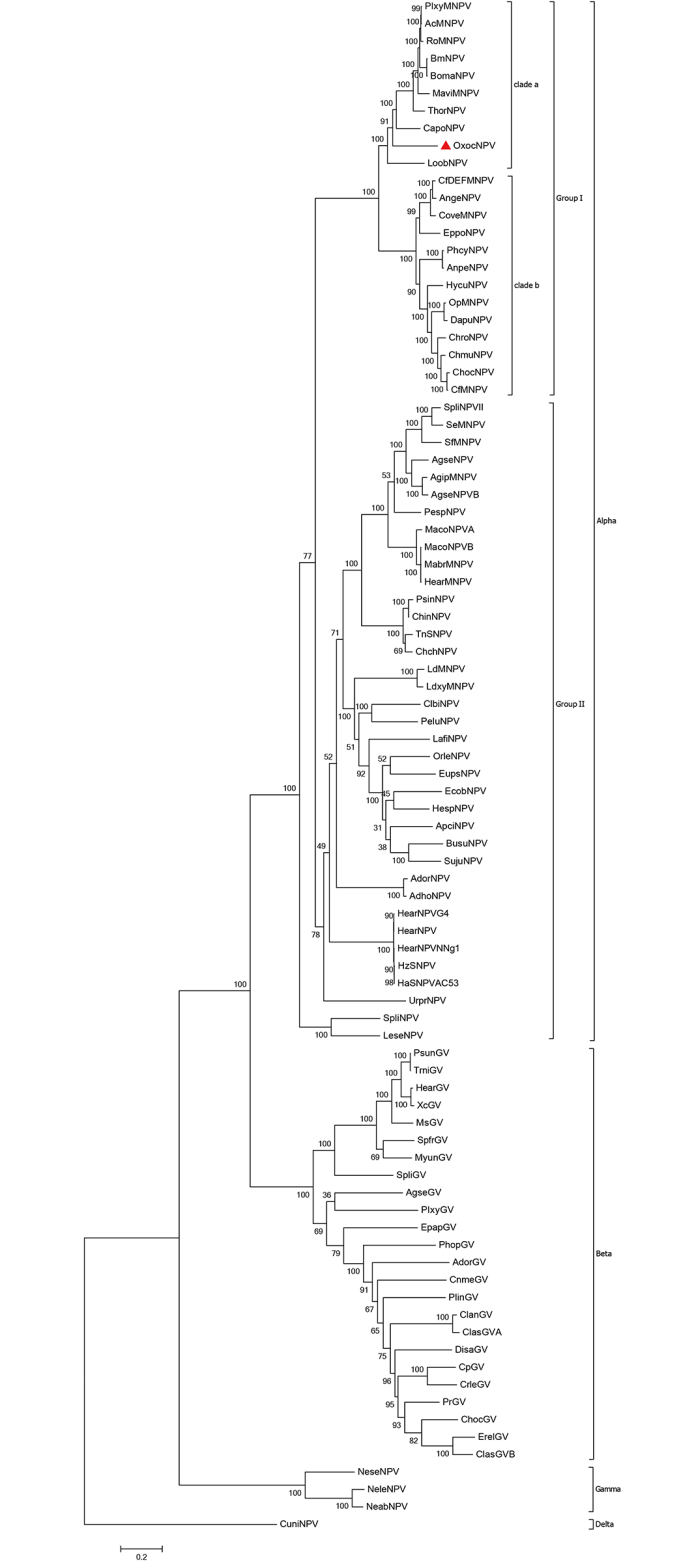
Phylogenic analysis of 88 complete baculovirus genomes. Phylogenetic analysis was performed using the combined aa sequences of the 38 core genes from 88 sequenced baculoviruses using the Maximum Likelihood method. Bootstrap value: 1000 replicates. The numbers on the nodes indicate the bootstrap scores. OxocNPV is indicated by a red triangle.

A phylogenetic tree based on 38 concatenated core genes from 88 whole-genome sequenced baculoviruses (including OxocNPV) was generated (Fig 2). According to the tree, OxocNPV can be classified as a new member of clade “a” within Group I alphabaculoviruses (indicated by a red triangle). It is located on a distinct branch and appears to be a close lineage to the most recent common ancestor of the Group I alphabaculoviruses.

### Relationship with other baculoviruses

Nine representative baculoviruses (either type species/well-studied representatives or in a close phylogenetic relation to OxocNPV) were chosen for the comparative study of OxocNPV: Autographa californica MNPV (AcMNPV, clade “a” Group I alphabaculovirus), Bombyx mori NPV (BmNPV, clade “a” Group I alphabaculovirus), Thysanoplusia orichalcea NPV (ThorNPV, clade “a” Group I alphabaculovirus), Catopsilia pomona NPV (CapoNPV, clade “a” Group I alphabaculovirus), Orgyia pseudotsugata MNPV (OpMNPV, clade “b” Group I alphabaculovirus), Helicoverpa armigera NPV (HearNPV, Group II alphabaculovirus), Cydia pomonella GV (CpGV, betabaculovirus), Neodiprion sertifer NPV (NeseNPV, gammabaculovirus) and Culex nigripalpus NPV (CuniNPV, deltabaculovirus). OxocNPV shares 119 homologous ORFs with AcMNPV, 115 with BmNPV, 114 with ThorNPV, 112 with CapoNPV, 112 with OpMNPV, 97 with HearNPV, 67 with CpGV, 49 with NeseNPV and 39 with CuniNPV. Regarding the 38 core genes, OxocNPV shares an average aa identity of 63.8%, 64.4%, 64.4%, 63.1%, 57.3%, 43.1%, 31.0%, 26.0%, 18.3% with the above nine viruses, respectively (S1 Table). When compared with the five selected Group I alphabaculoviruses (AcMNPV, BmNPV, ThorNPV, CapoNPV and OpMNPV), OxocNPV shares an average aa identity of 58.1%, 51.8%, and 38.3% for the core genes, lepidopteran baculovirus conserved genes and other baculoviral genes, respectively (S2 Table). Eight OxocNPV ORFs share high homology (>75% aa identity on average) with their counterparts in the other five selected Group I alphabaculoviruses, and the majority of them are core genes/lepidopteran baculovirus conserved genes, except *ubiquitin* (S2 Table). In contrast, among the 14 less conserved ORFs (<30% aa identity in average), only one core gene (*desm oplakin*) and one lepidopteran baculovirus conserved gene (*lef6*) were found (S2 Table).

**Fig 3:**
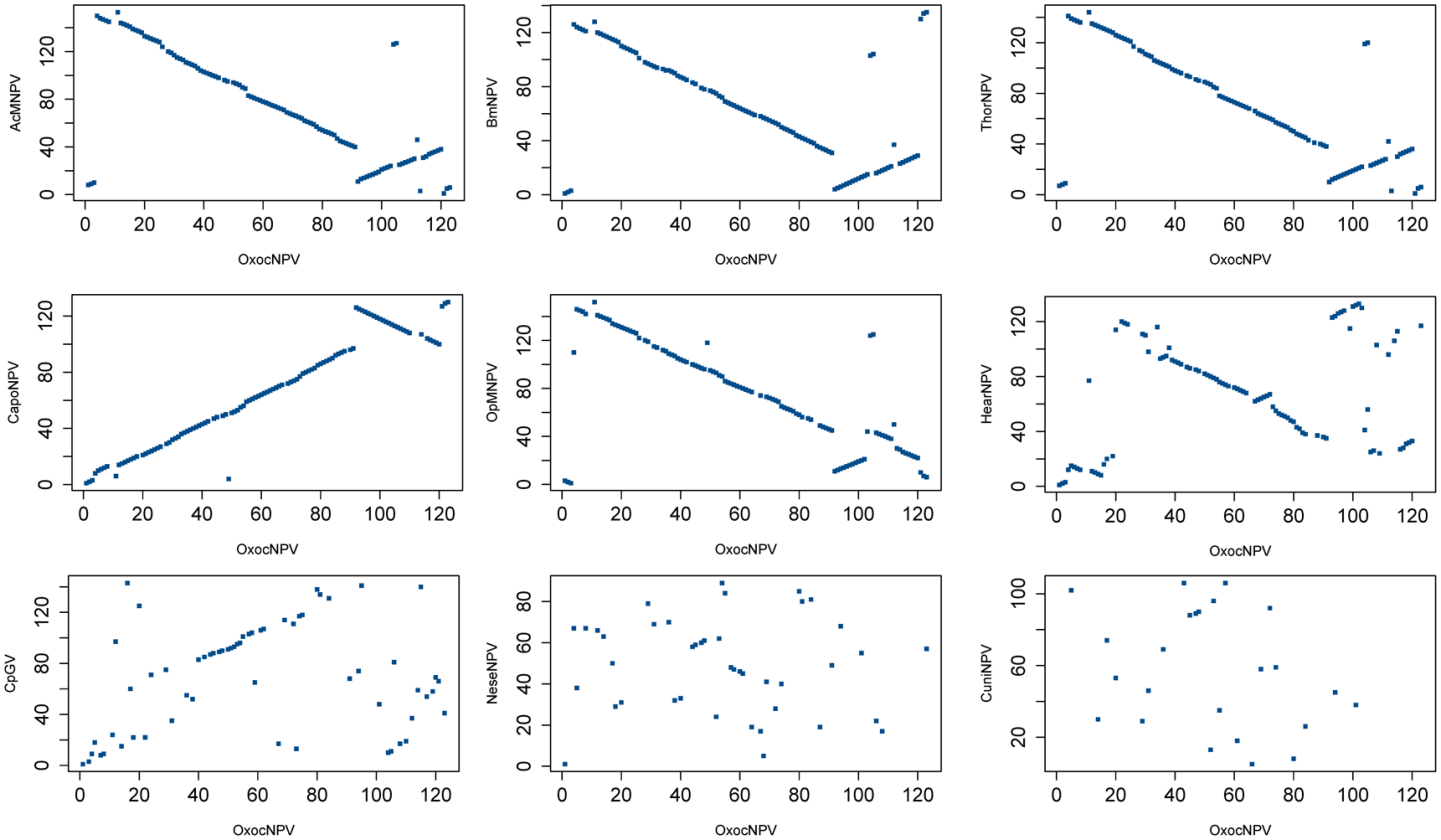
Gene parity plot analysis. Gene parity plots were constructed of OxocNPV against representative baculoviruses: AcMNPV, BmNPV, ThorNPV and CapoNPV (Group I clade “a”); OpMNPV (Group I clade “b”); HearNPV (Group II); CpGV (a betabaculovirus), NeseNPV (a gammabaculovirus) and CuniNPV (a deltabaculovirus). OxocNPV ORFs are on the x-axes. The accession numbers of these genomes are listed in S1 Table.

Gene parity plots of OxocNPV against the above nine selected baculoviruses are shown in Fig 3. The OxocNPV gene order is substantially collinear with representatives of both Group I and Group II alphabaculoviruses, with a small region that is collinear with betabaculoviruses; however, the gene order is significantly different from that of gamma- and deltabaculoviruses (Fig 3). The previously identified collinear region, which is conserved among lepidopteran baculoviruses and characterized by containing a highly similar gene contents (harboring ~20 core genes) and gene orders [19] is also conserved in OxocNPV. In OxocNPV, this region contains 20 core genes, 5 lepidopteran baculovirus conserved genes and 8 other baculoviral genes (Fig 1).

### Homologous regions

**Fig 4:**
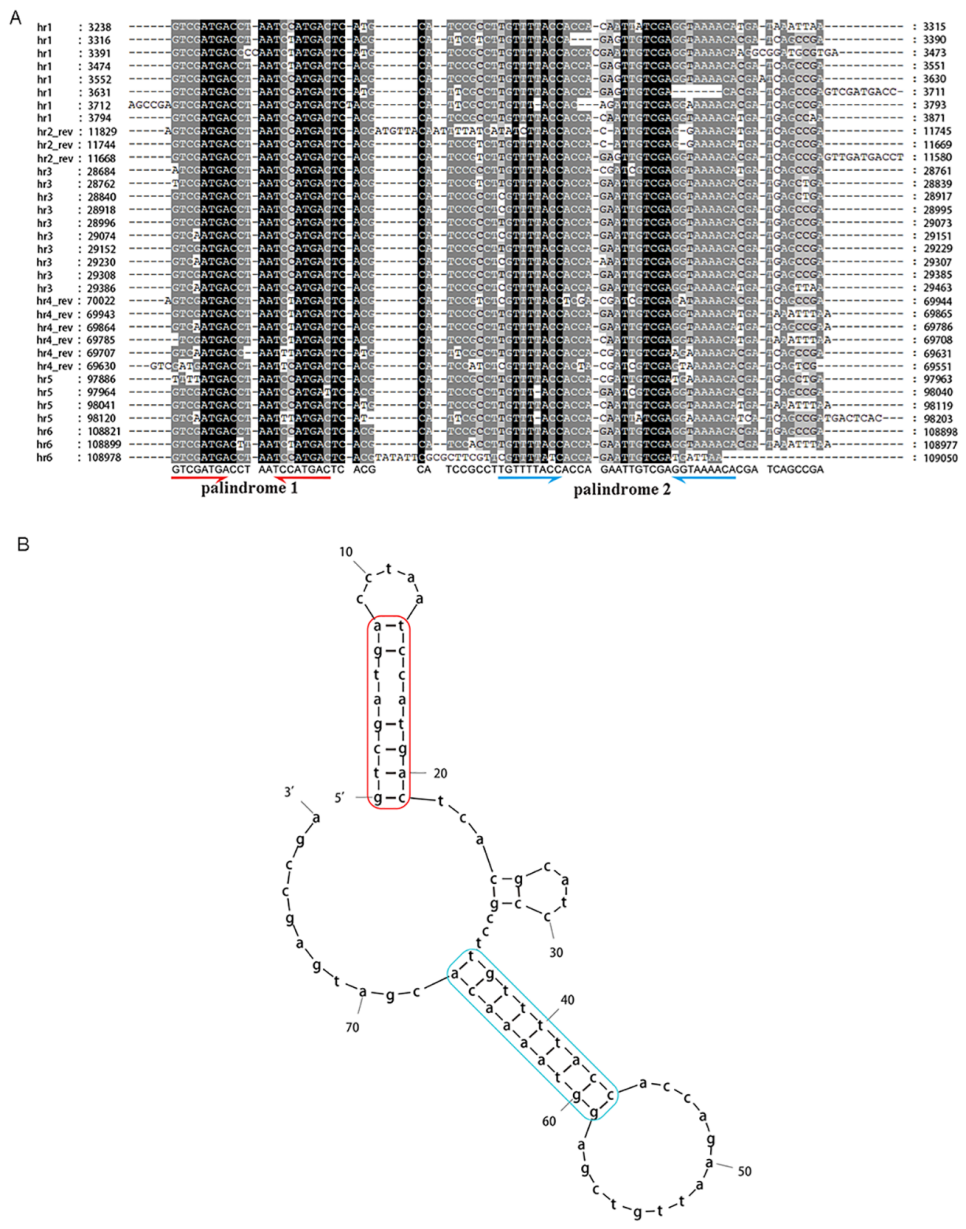
Analyses of the sequence and secondary structure of OxocNPV *hrs*. (A) Sequence alignment of OxocNPV *hr*s. Black background indicates 100% identity among compared regions, and dark and light gray indicates >80% and >60% identity, respectively. The numbers of *hrs* are listed on the left, and the conserved sequence and two palindromes are listed at the bottom. (B) Secondary structure prediction of conserved *hr* sequences. The red rectangle indicates the first palindrome and the blue rectangle indicates the second palindrome.

A typical characteristic of baculovirus genomes is the presence of interspersed homologous regions (*hrs*) with high A+T content, tandem repeats and imperfect palindromes, although they do not necessarily exist in all baculoviruses [20, 21]. *Hrs* have been implicated both as origins of DNA replication and as transcriptional enhancers in a number of baculoviruses [22, 23]. Six *hrs* were found in the OxocNPV genome, and they had an A+T content of 54.3% (Fig 1, pink, and Fig 4). *Hr2* and *hr4* are positioned in a counterclockwise direction and the rest are positioned in a clockwise direction in the genome. The length of the OxocNPV *hrs* ranges from 230–780 bp, and each *hr* consists of tandem repeats of about 80 bp in length (Fig 4). The secondary structure prediction of the tandem repeats revealed that it contains two imperfect palindromes (Fig 4, red and blue, respectively).

### Gene content of OxocNPV

Annotation of the OxocNPV genome revealed that it contains 11 replication-associated genes, 12 transcription-associated genes, 34 structure-related genes, 10 genes essential for oral infection, and 20 auxiliary genes (Table 1). In addition, 37 genes of unknown function including three hypothetical unique OxocNPV genes were predicted (Table 1).

**Table 1:** Gene contents of OxocNPV*.

Gene type	Core genes	Lepidoptera baculovirus conserved genes	Other baculoviral genes
**Replication**	*alk-exo* (*oxoc20*), *helicase* (*oxoc48*), *dna-pol* (*oxoc72*), *lef1* (*oxoc95*), *lef2* (*oxoc124*)	*ie-1* (*oxoc6*), *me53* (*oxoc16*), *lef3* (*oxoc70*), *dbp* (*oxoc107*), *lef11* (*oxoc120*)	*ac79* (*oxoc59*)
**Transcription**	*lef5* (*oxoc44*), *lef4* (*oxoc53*), *vlf-1*(*oxoc61*), *lef9* (*oxoc74*), *lef8* (*oxoc85*), *p47* (*oxoc92*)	*pk-1* (*oxoc3*), *lef6* (*oxoc110*), *39k* (*oxoc119*),	*pe38* (*oxoc11*), *lef10* (oxoc81), *lef12* (*oxoc91*)
**Structure**	*odv-ec27* (*oxoc12*), *odv-e18* (*oxoc13*), *49k* (*oxoc14*), *odv-ec43* (*oxoc36*), *p48/p45* (*oxoc40*), *p40* (*oxoc42*), *p6*.*9* (*oxoc43*), *38k* (*oxoc45*), *odv-e25* (*oxoc50*), *p18* (*oxoc51*), *p33* (*oxoc52*), *vp39* (*oxoc54*), *ac81* (*oxoc57*), *gp41* (*oxoc58*), *ac78* (*oxoc60*), *desmoplakin* (*oxoc71*), *vp1054* (*oxoc80*), *ac53* (*oxoc82*)	*polyhedrin* (*oxoc1*), *p12* (*oxoc41*), *tlp-20* (*oxoc56*), *fp25k* (*oxoc75*), *F* (*oxoc103*), *calyx/pep* (*oxoc22*)	*p78/83* (*oxoc2*), *exon0* (*oxoc15*), *p10* (*oxoc18*), *p24* (*oxoc24*), *gp16* (*oxoc23*), *gp64* (*oxoc25*), *vp80* (*oxoc39*), *odv-e26* (*oxoc97*), *pkip* (*oxoc104*)
**Oral infection**	*pif5* (*oxoc5*), *p74* (*oxoc17*), *pif1* (*oxoc29*), *pif3* (*oxoc31*), *pif4* (*oxoc47*), *vp91/p95* (*oxoc55*), *pif6* (*oxoc69*), *pif2* (*oxoc102*), *ac110* (*oxoc35*)	*ac108* (*oxoc37*)	*odv-e66* (*oxoc113*)
**Auxiliary**		*38*.*7k* (*oxoc94*), *ADPRase* (*oxoc121*),	*p26* (*oxoc19*), *iap-2* (*oxoc67*), *MTase* (*oxoc68*), *gp37* (*oxoc73*), *bjdp* (*oxoc84*), *trax-like* (*oxoc86*), *gta* (*oxoc90*), *egt* (*oxoc96*), *arif-1* (*oxoc101*), *chitinase* (*oxoc105*), *cath* (*oxoc106*), *iap-1* (*oxoc109*), *ac30* (*oxoc112*), *ctl* (*oxoc114*), *sod* (*oxoc115*), *fgf* (*oxoc116*), *ubiquitin* (*oxoc118*), *ptp* (*oxoc122*)
**Unknown**		*ac146* (*oxoc7*), *ac145* (*oxoc8*), *ac106* (*oxoc38*), *ac76* (*oxoc62*), *ac75* (*oxoc63*)	*ac150* (*oxoc4*), *ac132* (*oxoc21*), *ac124*(*oxoc26*), *ac120* (*oxoc28*), *ac117* (*oxoc30*), *ac114* (*oxoc32*), *ac113* (*oxoc33*), *ac111* (*oxoc34*), *oxoc46*, *oxoc49*, *ac74* (*oxoc64*), *ac73* (*oxoc65*), *ac72* (*oxoc66*), *ChaB-like* (*oxoc76*), *ac59* (*oxoc77*), *ac57* (*oxoc78*), *ac55* (*oxoc79*), *ac52* (*oxoc83*), *ac45* (*oxoc87*), *ac44* (*oxoc88*), *ac43* (*oxoc89*), *ac11* (*oxoc93*), *ac17* (*oxoc98*), *ac18* (*oxoc99*), *ac19* (*oxoc100*), *ac26* (*oxoc108*), *ac29* (*oxoc111*), *ac34* (*oxoc117*), *ac5* (*oxoc123*)

*The hypothetical unique genes (*oxoc9*, *oxoc10*, *ox oc27*) of OxocNPV are not included.

### DNA replication and RNA transcription genes

**Fig 5:**
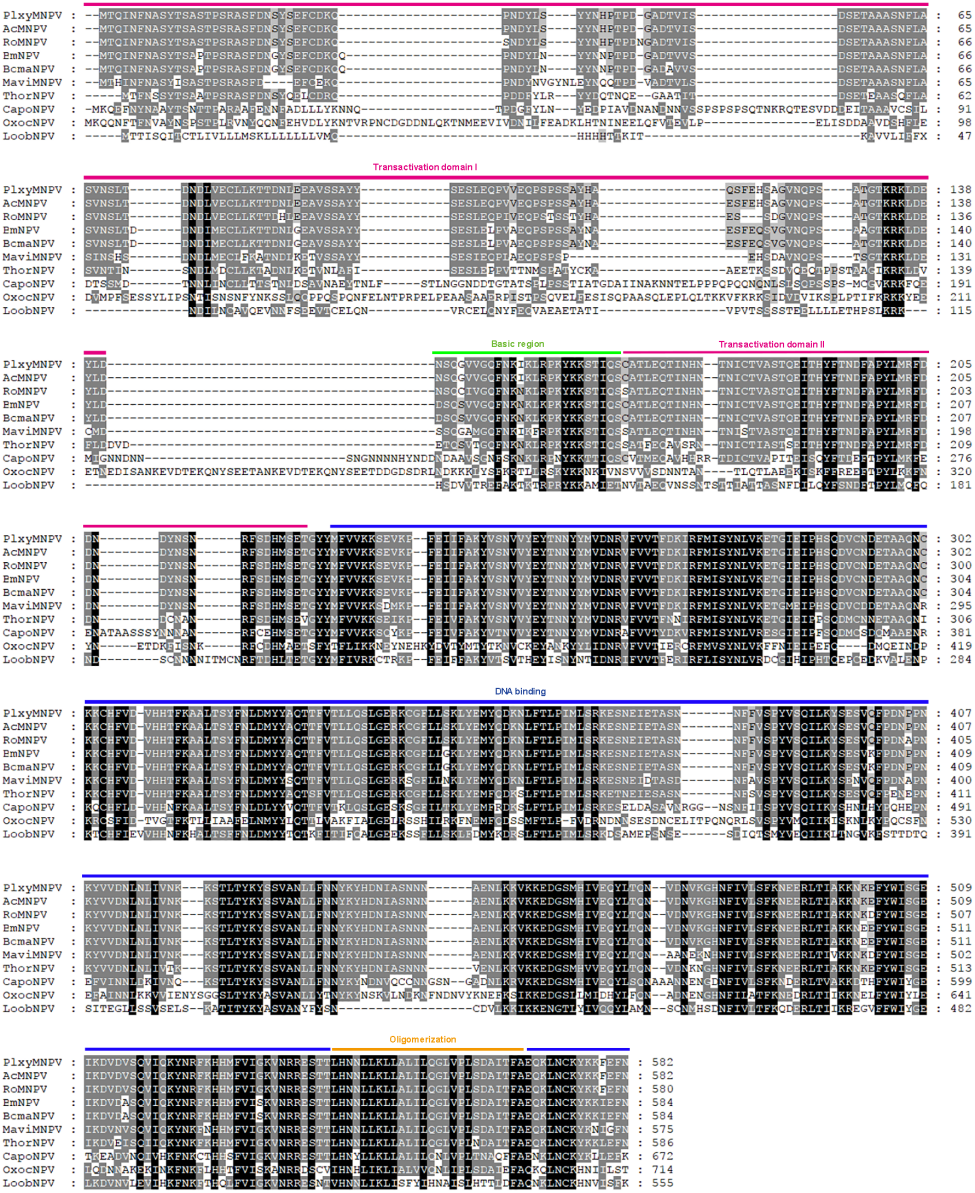
Sequence alignment of IE-1 from clade “a” of Group I baculoviruses. Functional domains were annotated according studies of AcMNPV IE-1. The red, green and orange lines indicate the transcriptional transactivation domains, basic region and oligomerization domain, respectively. The putative DNA-binding domain is indicated by the blue line. The GenBank accession numbers for these IE-1 proteins and the virus full names are as follows: YP_758611 (Plutella xylostella multiple nucleopolyhedrovirus, PlxyMNPV), NP_054178 (AcMNPV), NP_703134 (RoMNPV), NP_047544 (BmNPV), YP_002884369 (Bombyx mandarina nucleopolyhedrovirus, BomaNPV), YP_950845 (Maruca vitrata nucleopolyhedrovirus, MaviNPV), YP_007250550 (ThorNPV), YP_009255268 (CapoNPV) and AKN80956.1(LoobNPV).

So far, six genes have been found to be essential for baculovirus DNA replication and they are all present in the OxocNPV genome: immediate early gene-1 (*ie-1*, *oxoc6*), DNA polymerase (*DNA-pol*, *oxoc72*), *helicase* (*oxoc48*), *late expression factor 1 (lef1*, *oxoc95*), *lef2* (*oxoc124*) and *lef3* (*lef3*, *oxoc70*) (Table 1) [24, 25]. Among them, IE-1, a major transcriptional activator of early genes, was found to be significant longer for OxocNPV (~714 aa) than for most of the other Group I alphabaculoviruses (~550 aa). Functional analysis showed that the N-terminal half of AcMNPV IE-1 contains two independent transcription stimulatory (transactivation) domains (M1-N125 and A168-G222) interrupted by a basic region, while the C-terminal half contains putative DNA-binding and oligomerization domains (Fig 5) [26, 27]. Sequence alignment showed that the transactivation domain I of OxocNPV IE-1 is quite divergent from the homologs in other clade “a” Group I alphabaculoviruses in that the OxocNPV domain contains several discontinuous insertions (Fig 5). Interestingly, IE-1 of CapoNPV and Lonomia obliqua multiple nucleopolyhedrovirus [LoobNPV], which are also closely related to the ancestral Group I alphabaculovirus (Fig 2), also exhibits obvious differences in this domain compared to other Group I alphabaculoviruses (Fig 5) [27]. Whether the transactivation domain I of OxocNPV, CapoNPV or LoobNPV IE-1 is sufficient to activate transcription (like its counterpart in AcMNPV IE-1) remains to be investigated, and this may provide useful information regarding the function–evolution relationship of the baculovirus IE-1 protein.

Additional genes that influence DNA replication were found in OxocNPV: *DNA binding protein* (*dbp*, *oxoc107*), *lef11* (*oxoc120*), *me53* (*oxoc16*), *alkaline exonuclease* (*alk-exo*, *oxoc20*) and *ac79* (*oxoc59*) [16] (Table 1). However, homologs of *lef7* (*ac125*) and proliferating cell nuclear antigen (*pcna*, *ac49*) were absent. Eukaryotic PCNA plays a role in DNA synthesis, DNA repair and cell cycle progression. In AcMNPV, PCNA was not found to play an obvious role in transient DNA replication [28]. However, it was found to accelerate expression of late genes [29]. LEF7 is a stimulating factor for viral DNA replication and it has been proposed to be a single-stranded DNA-binding protein [30, 31]. Deletion of AcMNPV *lef7* resulted in a >90% reduction in viral DNA replication in Sf21 and SE1c cells, but not in Tn368 cells [32]. Deletion of *lef7* from the BmNPV genome also led to impairment of viral DNA synthesis [33]. Recent study suggested that AcMNPV LEF7 promoted efficient virus replication most likely by hijacking host factors regulating the DNA damage response [34]. So far, homologs of *lef7* have been found to be present in all the sequenced Group I alphabaculoviruses except OxocNPV, CapoNPV and LoobNPV [35].

Early baculovirus genes are transcribed by the host cell RNA polymerase II, but after onset of DNA replication, the transcription of late and very late genes is dependent on viral-encoded RNA polymerase, a 560-kDa protein complex composed of LEF-4, 8, 9 and P47 [36]. In the OxocNPV genome, six core genes (the four components of RNA polymerase plus *lef5* [*oxoc44*], and *very late factor 1* [*vlf-1*, *oxoc61*]), three lepidopteran baculovirus conserved genes and two other baculoviral genes related to viral late gene transcription were identified (Table 1) [36, 37]. OxocNPV *lef6*, CapoNPV *lef6* and LoobNPV *lef6* share low similarity (~20–40% aa identity) with other members of Group I clade “a”. LEF6 is required for late gene transcription and may function as an mRNA exporter. Deletion of AcMNPV *lef6* leads to a ~90% reduction in infectious budded virus (BV) production [38]. The homolog of *ie-2*, a specific gene of Group I alphabaculoviruses, is absent from the genome of OxocNPV. IE-2 contains a predicted really interesting new gene (RING) finger domain and has been found to enhance transactivation when acting synergistically with IE-1 [39–41]. Deletion of *ie-2* reduced the plasmid replication level by 3-fold in Sf21 cells [42]. OxocNPV is the first reported Group I alphabaculovirus to lack *ie-2*.

### Structural genes

Eighteen core genes and six lepidopteran conserved genes that encode structural proteins were identified in the OxocNPV genome (Table 1) [43–45]. In addition, nine other baculoviral genes were also identified in the OxocNPV genome (Table 1). *Desmoplakin* (*ac66*) is one of the 38 core genes. Knockout of *ac66* led to a >99% reduction in BV yield compared to the wild-type virus, as well as the elimination of occlusion-derived virus (ODV) and OB formation [46]. Certain baculoviruses harbor two or three copies of *desmoplakin*. In the OxocNPV genome, only one *desmoplakin* gene (*oxoc71*) is present and its protein length (683 aa) is much shorter than those of other Group I homologs (766–953 aa), due to many deletions in the middle region (S1 Fig, only clade “a” members are shown). *Cg30* is present in the genomes of most sequenced alphabaculoviruses and certain betabaculoviruses (such as SpliGV). Regarding Group I alphabaculoviruses, *cg30* is missing only in two cases, OxocNPV and Maruca vitrata multiple nucleopolyhedrovirus (MaviNPV). CG30 contains putative RING finger and leucine zipper domains. It is not an essential gene for AcMNPV replication as deletion of *cg30* resulted in only a subtle reduction in the BV titer [47]. In a study of BmNPV, CG30 was found to be required for maximum BV production and OB formation [48]. Therefore, the acquisition of *cg30* may represent a selective advantage during evolution.

### Proteins involved in primary infection

*Per os* infectivity factors (PIFs) are a group of ODV-specific envelope proteins that are required for the establishment of primary infection [49, 50]. So far, all ten recognized PIF genes have been found in the OxocNPV genome, comprising *p74* (*oxoc17*), *pif1* (*oxoc29*), *pif2* (*oxoc102*), *pif3* (*oxoc31*), *pif4* (*oxoc47*), *pif5* (*oxoc5*), *pif6* (*oxoc69*), *pif7 (ac110*, *oxoc35*), *pif8* (*vp91/p95*, oxoc55) and *sf58* (*oxoc37*) (Table 1; S1 Table) [51–54]. Except for the last PIF gene, which is a lepidopteran baculovirus conserved gene, the PIF genes are core genes [55].

Besides PIFs, other ODV envelope proteins also play important roles in *per os* infection. ODV-E66 (*ac46*) is also a major component of ODV envelope proteins. Homologs of *odv-e66* are found in the genomes of most alpha- and betabaculoviruses, but not in gamma- or deltabaculoviruses. Deletion of AcMNPV *odv-e66* resulted in a 1000-fold increase in the lethal dose that kills 50% of a test sample (LD_50_) compared to wild-type virus when larvae were infected *per os*, but there was no difference when the virus was injected into the hemolymph [56]. Recently, ODV-E66 was shown to have chondroitinase activity and it has been suggested that it facilitates the primary infection of ODV by digestion of chondroitin sulfate in the insect midgut peritrophic membrane [57].

**Fig 6:**
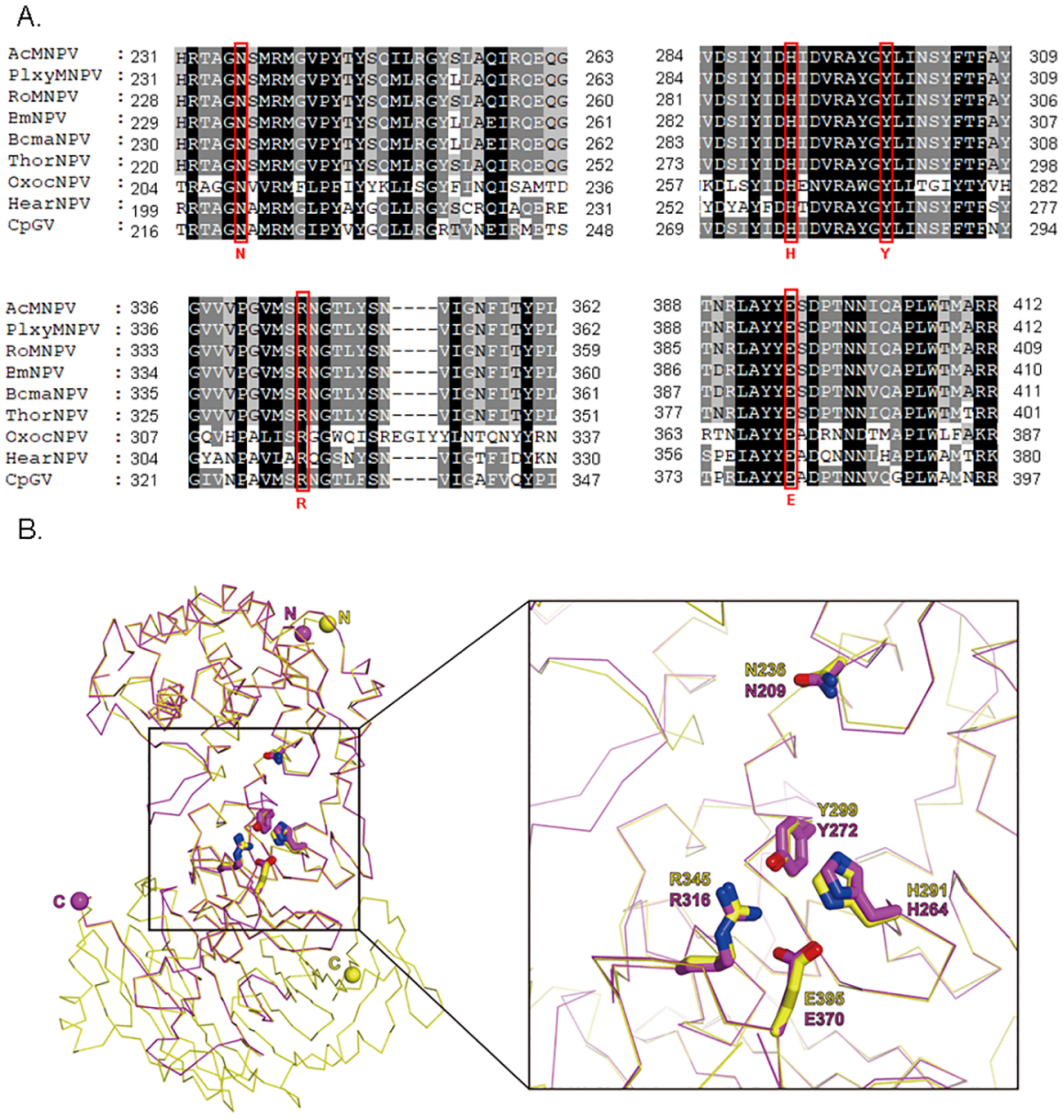
Conservation of key active residues in ODV-E66. (A) Sequence alignment of OxocNPV ODV-E66 with the sequences of eight representative baculoviruses. The five key active residues are marked by rectangles. The GenBank accession numbers for these ODV-E66 proteins and the virus full names are as follows: NP_054075 (AcMNPV), YP_758513 (PlxyNPV), NP_703036 (RoMNPV), NP_047452 (BmNPV), YP_002884277 (BomaNPV), YP_007250454 (ThorNPV), NP_203651 (HearNPV) and NP_148821 (CpGV). (B) Prediction of the 3D structure of OxocNPV ODV-E66. The computational 3D structure (red) of OxocNPV ODV-E66 was modeled using the crystal structure (yellow) of AcMNPV ODV-E66 (PDB code, 3vsm) as the template [58]. The five key active residues of OxocNPV ODV-E66 (N209, H264, Y272, R316 and E370) were superimposed over the corresponding residues of AcMNPV ODV-E66 (N236, H291, Y299, R345 and E395). The image on the right represents the enlarged active site of the left image.

Among the 23 sequenced Group I alphabaculoviruses, all encode odv-e66 except MaviNPV, CapoNPV and Condylorrhiza vestigialis MNPV [CoveNPV]). Interestingly, sequence alignment showed that OxocNPV ODV-E66 (*oxoc113*) shares low amino acid serquence identity (~25%) with the ODV-E66 sequences of other Group I members. The Group I homologs ODV-E66 normally exhibit a high degree of sequence conservation (>70% identity) (S2 Fig). It appears that the N-terminus of OxocNPV ODV-E66, which contains a polysaccharide lyase family sequence with homology to bacterial chondroitinases, is more conserved than its C-terminus, which is a baculovirus ODV-E66 superfamily domain with unknown function (S2 Fig) [58,59]. Five residues were identified as being essential for the catalytic actively of AcMNPV ODV-E66 [58], and these residues were also conserved in the protein of OxocNPV (Fig 6A).

The three-dimensional (3D) structure of OxocNPV ODV-E66 (Fig 6B, magenta) was modeled based on the reported AcMNPV ODV-E66 crystal structure (Fig 6B, yellow, Protein Data Bank (PDB) code: 3vsm) [58], and the superimposition of these two structures revealed a higher degree of overlap in the N-terminal α-helix-rich domain compared to the C-terminal β-strand-rich region (Fig 6B, left figure). The five key active residues of OxocNPV ODV-E66 (N209, H264, Y272, R316 and E370) were suitably superimposed on the corresponding residues of AcMNPV ODV-E66 (N236, H291, Y299, R345 and E395) (Fig 6B, right image).

These findings suggest that OxocNPV may encode an active ODV-E66, although further investigation is required. The significant difference in ODV-E66 between OxocNPV and other Group I members also suggests a more ancient origin of OxocNPV during the evolution of the Group I lineage.

### Auxiliary genes

Auxiliary genes are not essential for viral replication, but they may provide a selective advantage to facilitate virus production/survival [60]. Homologues of auxiliary genes in OxocNPV include but are not limited to *38*.*7k* (*oxoc94*), *ADP-ribose pyrophosphatase* (*ADPRase*, *oxoc121*), *ubiquitin* (*oxoc118*), *arif-1* (*oxoc101*), *cathepsin* (*oxoc106*), *chitinase* (*oxoc105*), *baculovirus J domain protein* (*bjdp*, *oxoc84*), *ecdysteroid UDP glucosyltransferase* (*egt*, *oxoc96*), *fibroblast growth factor* (*fgf*, *oxoc116*), *gp37* (*oxoc73*), *global transactivator* (*gta*, *oxoc90*), *p26* (*oxoc19*), *phosphotyrosine phosphatase* (*ptp*, *oxoc122*), *inhibitor of apoptosis-1* (*iap-1*, *oxoc109*), *iap-2* (*oxoc67*), *superoxide dismutase* (*sod*, *oxoc115*), *trax-like* (*oxoc86*), *MTase* (*oxoc68*), and *conotoxin-like* (*ctl*, *oxoc114*) (Table 1). However, baculovirus repeated ORFs (*bro*), which are repeated genes that are found in most baculoviruses and contain DNA-binding activity that could influence host DNA replication and transcription, were absent from the OxocNPV genome, which is similar to the situation in MaviNPV and Rachiplusia ou MNPV (RoMNPV) in the Group I lineage.

### Unique genes

Three ORFs of OxocNPV, comprising *oxoc9* (203 aa), *oxoc10* (79 aa) and *oxoc27* (154 aa), have no discernible homologues in GenBank. Interestingly, *oxoc9* and *oxoc10* are linked and their sequences have been validated by PCR. Whether these are functional ORFs of OxocNPV requires further experimentation.

## Materials and methods

### Viral DNA extraction

OxocNPV-infected *O*. *ochracea* larvae have been preserved at the Chinese General Virus Collection Center (CGVCC) under collection number IVCAS 1.0235. The virus OBs were purified from larvae body homogenate by differential centrifugation [61]. Viral genomic DNA was isolated according to the method reported previously [62, 63].

### Genomic DNA sequencing and bioinformatics analysis

Genomic DNA sequencing of OxocNPV was performed using the Roche 454 GS FLX pyrosequencing system. The sequenced reads were assembled with 454 Newbler software version 2.7. Low-quality regions or ambiguous bases were further verified by PCR and Sanger sequencing.

The establishment of the full genome sequence of OxocNPV was followed by ORF and repeated regions prediction. The *hrs* were determined using Tandem Repeats Finder (http://tandem.bu.edu/trf/trf.html) [64] and the NCBI BLAST server (http://blast.ncbi.nlm.nih.gov/Blast.cgi). Putative ORFs were predicted using FGENESV0 (http://linux1.softberry.com/berry.phtml) [65] and the NCBI ORF finder (http://www.ncbi.nlm.nih.gov/gorf/gorf.html), using the criteria of protein length >50 aa and minimal overlaps. The predicted ORFs were annotated according to homology using NCBI BLAST. The complete genome sequence and annotation information were submitted to GenBank (accession number: MF143631). Gene parity plots were constructed to compare ORF organization, as previously described [66].

### Phylogenetic analysis

The concatenated protein sequences encoded by the 38 core genes of OxocNPV and the other 87 sequenced baculovirus genomes were aligned using ClustalW [67]. A phylogenetic tree was reconstructed using the Maximum Likelihood method based on the Jones–Thornton–Taylor (JTT) model with 1000 bootstrap values for core proteins using MEGA6 software [68]. The reliability of the tree was explored via a bootstrap analysis with 1000 replicates [69].

### 3D structural modeling

The 3D structural model of OxocNPV ODV-E66 was predicted by the SWISS-MODEL server (http://www.expasy.org/structural_bioinformatics), using the AcMNPV ODV-E66 structure (PDB code, 3vsm) as the template. A superimposition of OxocNPV ODV-E66 and AcMNPV ODV-E66 was generated by PyMOL (www.pymol.org).

## Supporting information

S1 Fig Sequence alignment of desmoplakin of clade “a” of Group I alphabaculoviruses. Black background indicates 100% identity among compared regions, and dark and light gray indicates >80% and >60% identity, respectively. The GenBank accession numbers for these Desmoplakin proteins and the virus full names are as follows: YP_758533 (PlxyMNPV), NP_054096 (AcMNPV), NP_703056 (RoMNPV), NP_047470 (BmNPV), YP_002884295 (BomaNPV), YP_950780 (MaviNPV), YP_007250473 (ThorNPV), ANF29722 (CapoNPV), AKN81050 (LoobNPV).
(TIF)

S2 Fig Sequence alignment of ODV-E66 of Group I alphabaculoviruses. The Black background indicates 100% identity among compared regions, and dark and light gray indicates >80% and >60% identity, respectively. GenBank accession numbers for these ODV-E66 proteins and the virus full names are as follows: YP_758513 (PlxyMNPV), NP_054075 (AcMNPV), NP_703036 (RoMNPV), NP_047452 (BmNPV), YP_002884277 (BomaNPV), YP_007250454 (ThorNPV), YP_803443 (Anticarsia gemmatalis nucleopolyhedronvirus, AngeNPV), NP_932654 (Choristoneura fumiferana DEF multiple nucleopolyhedronvirus, CfDEFMNPV), NP_203211 (Epiphyas postvittana nucleopolyhedronvirus, EppoNPV), YP_611070 (Antheraea pernyi nucleopolyhedronvirus, AnpeNPV), AFY63904 (*Philosamia cynthia* nucleopolyhedronvirus, PhcyNPV), YP_473294 (Hyphantria cunea nucleopolyhedronvirus, HycuNPV), NP_046206 (OpMNPV), YP_008378455 (C. rosaceana nucleopolyhedroviruses, ChroNPV), YP_008992195 (Choristoneura murinana nucleopolyhedroviruses, ChmuNPV), NP_848356 (Choristoneura fumiferana MNPV, CfMNPV), YP_008378605 (Choristoneura occidentalis nucleopolyhedroviruses, ChocNPV), AKN81064 (Lonomia obliqua multiple nucleopolyhedrovirus), AKR14189 (Dasychira pudibunda nucleopolyhedrovirus).
(TIF)

S1 Table Genome annotation of OxocNPV. (XLSX)

S2 Table Conservation of OxocNPV genes compared to those of the five selected Group I alphabaculoviruses. (DOCX)
