# Supplementary figures and images for: Genome analysis of a novel Group I alphabaculovirus obtained from Oxyplax ochracea
Source: PLoS One. 2018 Feb 1;13(2):e0192279. doi: 10.1371/journal.pone.0192279 (PMC5794183; doi:10.1371/journal.pone.0192279)

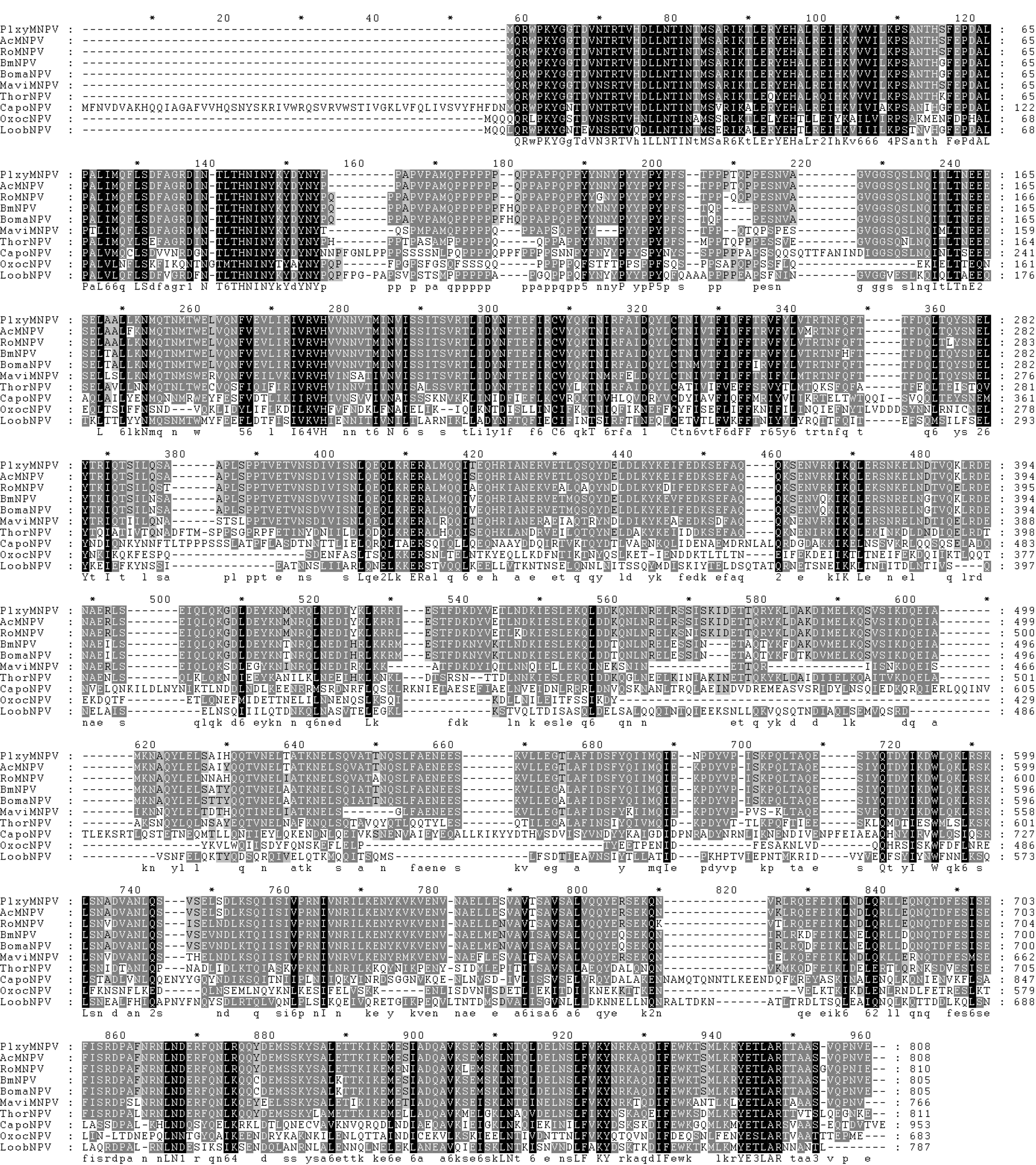

Supplement: S1 Fig — Black background indicates 100% identity among compared regions, and dark and light gray indicates >80% and >60% identity, respectively. The GenBank accession numbers for these Desmoplakin proteins and the virus full names are as follows: YP_758533 (PlxyMNPV), NP_054096 (AcMNPV), NP_703056 (RoMNPV), NP_047470 (BmNPV), YP_002884295 (BomaNPV), YP_950780 (MaviNPV), YP_007250473 (ThorNPV), ANF29722 (CapoNPV), AKN81050 (LoobNPV). (TIF) [file pone.0192279.s001.tif]

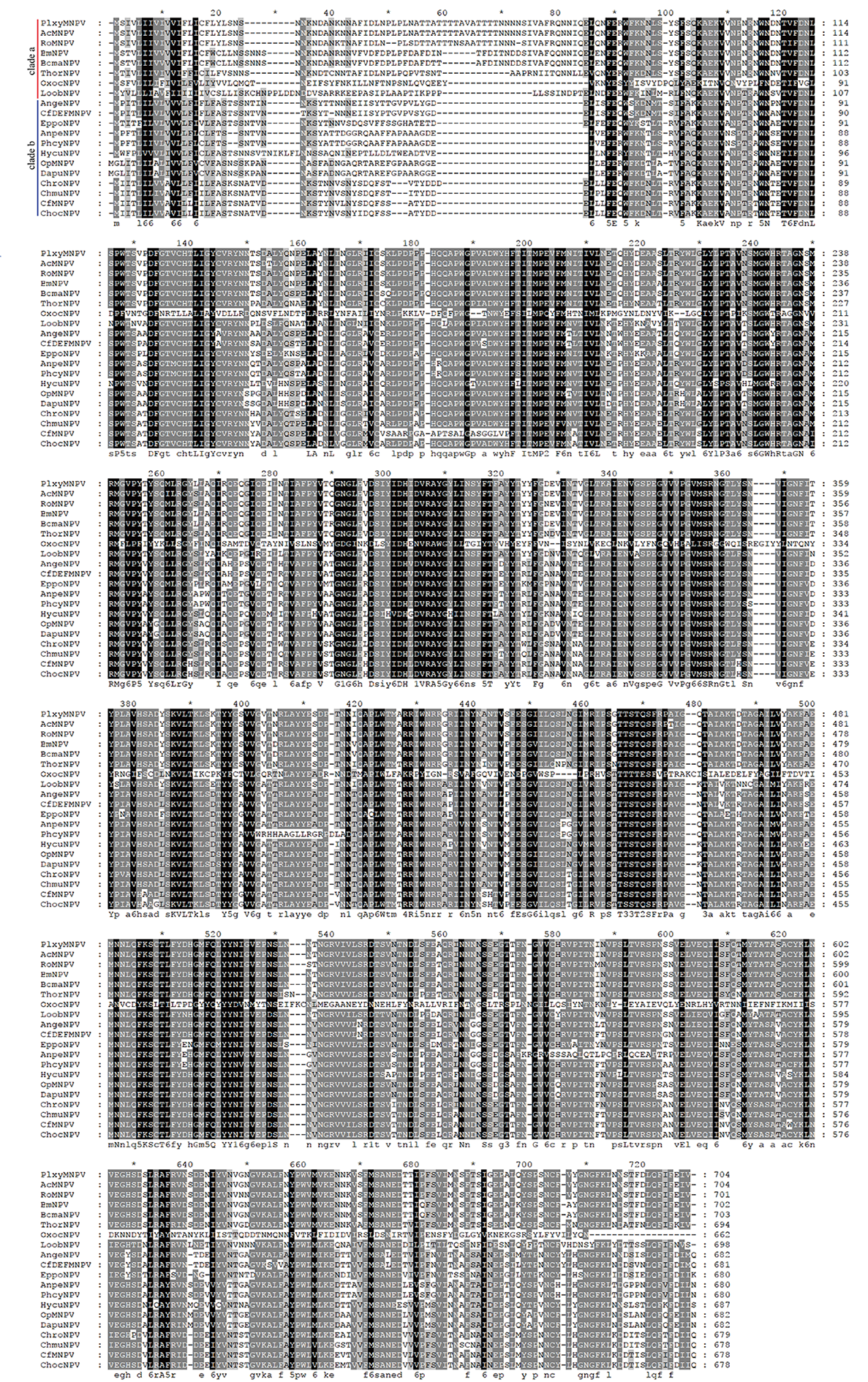

Supplement: S2 Fig — The Black background indicates 100% identity among compared regions, and dark and light gray indicates >80% and >60% identity, respectively. GenBank accession numbers for these ODV-E66 proteins and the virus full names are as follows: YP_758513 (PlxyMNPV), NP_054075 (AcMNPV), NP_703036 (RoMNPV), NP_047452 (BmNPV), YP_002884277 (BomaNPV), YP_007250454 (ThorNPV), YP_803443 (Anticarsia gemmatalis nucleopolyhedronvirus, AngeNPV), NP_932654 (Choristoneura fumiferana DEF multiple nucleopolyhedronvirus, CfDEFMNPV), NP_203211 (Epiphyas postvittana nucleopolyhedronvirus, EppoNPV), YP_611070 (Antheraea pernyi nucleopolyhedronvirus, AnpeNPV), AFY63904 (Philosamia cynthia nucleopolyhedronvirus, PhcyNPV), YP_473294 (Hyphantria cunea nucleopolyhedronvirus, HycuNPV), NP_046206 (OpMNPV), YP_008378455 (C. rosaceana nucleopolyhedroviruses, ChroNPV), YP_008992195 (Choristoneura murinana nucleopolyhedroviruses, ChmuNPV), NP_848356 (Choristoneura fumiferana MNPV, CfMNPV), YP_008378605 (Choristoneura occidentalis nucleopolyhedroviruses, ChocNPV), AKN81064 (Lonomia obliqua multiple nucleopolyhedrovirus), AKR14189 (Dasychira pudibunda nucleopolyhedrovirus). (TIF) [file pone.0192279.s002.tif]
