# Supplementary material for: Genome analysis of a novel Group I alphabaculovirus obtained from Oxyplax ochracea
Source: PLoS One. 2018 Feb 1;13(2):e0192279. doi: 10.1371/journal.pone.0192279 (PMC5794183; doi:10.1371/journal.pone.0192279)
Supplement: S2 Table — (DOCX) [file pone.0192279.s004.docx]

| Gene type | Average aa identity with 5 selected Group I alpabaculoviruses | > 75% | <30% |
| --- | --- | --- | --- |
| Core genes | 58.1% | *p33*(77.4%), *lef9*(77.2%), *pif2*(75.7%), *p74*(75.2%), *p18*(75.1%) | *desmoplakin*(26.4%) |
| Lepidoptera baculovirus conserved genes | 51.8% | *polyhedrin*(92.1%), ac76(77.0%) | *lef6* (29.9%) |
| Other baculoviral genes | 38.3% | *ubiquitin*(81.7%) | *pp78/83*(29.8%), *ac150*(29.6%), *ac45*(29.1%), *ac112/ac113*(28.2%), *odv-e66*(27.4%), *ac5*(26.9%), *arif-1*(26.6%), *oxoc46*(26.5%), *ac124*(25.8%), *ac132*(22.4%), *oxoc49*(22.2%), *pe38*(19.9%) |
